# Living with adenomyosis and involuntary childlessness: a qualitative interview study

**DOI:** 10.1186/s12905-026-04880-7

**Published:** 2026-09-14

**Authors:** Katharina Weiss, Annika Hiller, Niko Kohls, Karin Meissner

**Affiliations:** 1 Institute for Applied Health Sciences, Coburg University for Applied Sciences and Arts, Friedrich-Streib-Str. 2, Coburg, 96450 Germany; 2 Department of Health Promotion, Coburg University for Applied Sciences and Arts, Friedrich-Streib-Str. 2, Coburg, 96450 Germany

**Keywords:** Adenomyosis, Infertility, Involuntary childlessness, Coping, Mental health, Psychological distress

## Abstract

**Background:**

Adenomyosis is a chronic gynecological condition associated with pain, abnormal uterine bleeding, and impaired fertility. However, little is known about the psychosocial experiences and coping strategies of individuals with adenomyosis and involuntary childlessness. This study aimed to explore their experiences, perceived psychological burden, and coping strategies, while additionally incorporating the perspectives of gynecological specialists.

**Methods:**

This qualitative interview study included 20 individuals with a medically documented diagnosis of adenomyosis recruited across three German-speaking countries as well as five specialists in obstetrics and gynecology practicing in Germany. Semi-structured online interviews addressed diagnostic and healthcare experiences, information needs, psychological burden, and coping strategies. Data were analyzed using qualitative content analysis with summarizing and structuring approaches, complemented by thematic-descriptive analysis.

**Results:**

Participants described prolonged pathways to diagnosis, including experiences of misdiagnosis and perceived medical invalidation, as well as fragmented care, insufficient information, and limited psychosocial support. They reported substantial psychosocial burden in the context of chronic symptoms and involuntary childlessness. Coping strategies included self-care, boundary-setting, social support, stable relationships, psychotherapy, peer exchange, and maintaining a positive mindset. Feeling taken seriously by healthcare professionals and receiving a diagnosis were frequently described as important experiences in the coping process. Specialists provided complementary perspectives and reported structural barriers, including limited consultation time and insufficient reimbursement. Concomitant or suspected endometriosis was documented in a substantial proportion of participants, limiting the attribution of reported symptoms and burden specifically to adenomyosis.

**Conclusion:**

The study shows that individuals with adenomyosis and involuntary childlessness experience prolonged diagnostic delays, fragmented care, and significant psychosocial burden, underscoring the need for holistic, patient-centered care that integrates medical, psychosocial, and reproductive health needs. Given the qualitative and diagnostically heterogeneous sample, these findings should be considered hypothesis-generating and warrant further evaluation in larger, more diverse studies.

**Supplementary Information:**

The online version contains supplementary material available at https://doi.org/10.1186/s12905-026-04880-7.

## Introduction

An increasing number of individuals remain childless, and childlessness is not always a conscious decision and may have various underlying causes. Adenomyosis is one of several gynecological conditions associated with impaired fertility. It is characterized by the presence of endometrial glands and stroma within the muscular wall of the uterus (myometrium). Although its classification has long been debated, adenomyosis is increasingly recognized as a distinct clinical entity separate from endometriosis. It exhibits distinct molecular and epigenetic characteristics, including alterations in immune and adhesion molecules, cytokines, inflammatory mediators, cell proliferation, and apoptosis [1]. Nevertheless, adenomyosis and endometriosis share clinical features and frequently coexist; MRI-based studies have reported adenomyosis in approximately 70% of individuals with endometriosis [2]. Both are chronic, hormone-dependent inflammatory conditions [3], although their etiopathogenesis and the nature of their relationship remain incompletely understood [4–6].

Because uniform diagnostic criteria for adenomyosis were lacking for many years, its prevalence cannot be determined precisely. Reported prevalence estimates among women of reproductive vary widely, ranging from 5% to 70%, with estimates of 20%-35% reported in most studies [7]. Histological analyses of hysterectomy specimens have identified adenomyosis in approximately 20%-30% of cases [8].

Pain is among the most commonly reported symptoms of adenomyosis, affecting approximately 77% of patients [1]. Common manifestations include dysmenorrhea and dyspareunia [1, 2], as well as abnormal uterine bleeding, including menorrhagia, metrorrhagia, and hypermenorrhea [2]. At the same time, approximately one-third of affected individuals may remain asymptomatic [2].

Adenomyosis has also been associated with impaired fertility and adverse reproductive outcomes. Approximately 11%-12% of women with adenomyosis have been reported to experience infertility [9]. According to the WHO’s first global guideline on the prevention, diagnosis, and treatment of infertility, infertility is a disorder of the male and female reproductive systems defined as the failure to achieve pregnancy after 12 months of regular unprotected sexual intercourse [10]. In a meta-analysis, Younes and Tulandi found lower pregnancy and live birth rates and higher miscarriage rates among women with adenomyosis [11]. Similarly, Nirgianakis et al. reported lower pregnancy rates following assisted reproduction and higher miscarriage rates among affected individuals [12]. Proposed mechanisms linking adenomyosis to impaired fertility include altered uterine and tubal transport and impaired implantation processes [8]. Furthermore, adenomyosis has been associated with adverse pregnancy outcomes, including miscarriage, preterm birth, preeclampsia, fetal growth restriction, peripartum hemorrhage, the need for cesarean delivery, fetal malpresentation, premature rupture of membranes, and uterine rupture [13–16]. However, the mechanisms underlying these associations remain incompletely understood, and reproductive outcomes may be influenced by multiple coexisting factors, including endometriosis [11, 16].

Despite increasing clinical attention to adenomyosis, gaps in awareness, diagnosis, and patient information have been reported. Previous studies have described experiences of delayed recognition, insufficient information regarding the condition and available treatment options, and patients’ feeling that their symptoms were not adequately acknowledged [16–19]. Such experiences may add to the challenges of living with a chronic gynecological condition and navigating healthcare.

The psychosocial implications of adenomyosis remain comparatively under-researched. Chronic pain and abnormal uterine bleeding can affect daily functioning and quality of life, while involuntary childlessness, miscarriage, and unsuccessful fertility treatment may be accompanied by anxiety, depressive symptoms, social isolation, and feelings of loss of control [17, 18]. For individuals experiencing adenomyosis alongside an unfulfilled desire to have children, these challenges may coexist and interact.

The ways in which individuals manage stress and psychological strain are commonly described as *coping*. Coping encompasses the cognitive and behavioral strategies used to maintain or restore psychological balance under stressful conditions and to deal with difficult life circumstances [19, 20]. Coping strategies may vary depending on the specific stressor, individual resources, and situational context. Understanding coping in the context of adenomyosis and involuntary childlessness may therefore provide important insights into affected individuals’ psychosocial needs and potential areas for supportive care.

To date, research on adenomyosis has focused on clinical and diagnostic aspects, while coping and psychosocial experiences have received comparatively limited attention. To our knowledge, this is the first qualitative interview study to examine coping strategies and psychosocial experiences among individuals with adenomyosis and involuntary childlessness. Following a phenomenological approach, the study focused on participants lived experiences in this context without seeking to establish a causal relationship between adenomyosis, involuntary childlessness, and the reported psychosocial burden. In line with the psychosocial focus of the study, involuntary childlessness was operationalized as an unfulfilled desire to have children, rather than a medical diagnosis of infertility. By exploring the participants’ experiences in depth, the study aimed to provide an empirical starting point for the development of targeted, evidence-based psychosocial support services, an area that has so far received limited attention in adenomyosis research and clinical care.

The study aimed to address the following research questions (RQ):

- RQ1: How much time elapses between the onset of symptoms reported by participants and the diagnosis of adenomyosis?
- RQ2: How often do affected individuals take the initiative themselves to seek comprehensive diagnostic evaluation, for example because gynecologists are insufficiently aware of the condition?
- RQ3: Were affected individuals sufficiently informed about adenomyosis and its potential impact on fertility at the time of diagnosis, regardless of an existing desire to have children?
- RQ4: Were resources and support services for managing both adenomyosis and involuntary childlessness introduced, offered, or referred to, and if so, by whom?
- RQ5: What are specialists’ perceptions of gynecologists’ awareness of adenomyosis and its potential effects on fertility?
- RQ6: What coping strategies do individuals with adenomyosis use in the context of their chronic condition and involuntary childlessness?
- RQ7: How do individuals with adenomyosis cope with the challenges they experience in the context of their chronic condition and involuntary childlessness?
- RQ8: What challenges do individuals with adenomyosis experience in the context of their chronic condition and involuntary childlessness?

The findings of this study are intended to provide an empirical basis for the development of appropriate outpatient psychosocial interventions for individuals with adenomyosis and involuntary childlessness, with the broader aim of supporting coping skills and promoting patient-centered care.

## Materials and methods

### Study design

This study explored the individual experiences and perceptions of women with adenomyosis and involuntary childlessness. Given the limited existing research and the complexity of the topic, a qualitative approach using semi-structured interviews was chosen to gain an in-depth understanding of the participants lived experiences within this context, without seeking to establish causal relationships between adenomyosis and the experiences reported.

The reporting of this study follows the COREQ guidelines (*Consolidated Criteria for Reporting Qualitative Research;* see electronic supplement 3*)* [21].

Ethical approval was obtained from the Ethics Committee of Coburg University prior to data collection (HC-Weiss-Meissner-Kohls-19112024).

Throughout the research process, the principles of good scientific practice [22] and ethical standards of the Declaration of Helsinki [23] were strictly observed.

All participants received comprehensive written information about the study procedures, aims, and conditions of participation prior to inclusion in the study. Written informed consent was obtained from all participants before data collection commenced.

### Recruitment of study participants

The study sample consisted of individuals with a uterus aged 18 years or older with a documented diagnosis of adenomyosis, classified according to the ENZIAN [24] or #ENZIAN [25] classification system or documented using the ICD-10 code N80.0 [26]. Prior to inclusion, the research team reviewed diagnostic documentation (e.g., physician’s letters or medical reports) for all participants to verify that the eligibility criterion of a documented adenomyosis diagnosis was met. Thus, eligibility was not based on participant self-report alone. Participants were recruited throughout the DACH region (Germany, Austria, Switzerland) via endometriosis centers, fertility clinics, prenatal counseling centers, public health departments, women’s health centers, and endometriosis associations. Recruitment materials were distributed through flyers, websites, newsletters, support groups, and social media platforms. All individuals who responded on their own initiative agreed to participate, and met the inclusion criteria, were interviewed. No preselection was conducted, to include as broad a range of participants as possible. Supplementary, gynecologists in Germany with expertise in endometriosis and adenomyosis working in outpatient or private practice settings were contacted directly and invited to participate in an interview.

Before participation, all affected individuals and gynecologists received detailed information regarding the objectives and background of the research project.

### Data collection

Semi-structured interview guides were developed separately for patients and gynecologists (see electronic supplement 1) and were pilot tested in advance using two test interviews. The data collection took place from March to July 2025. During the patient interviews, 11-points numeric rating scales (NRS) (0 = no burden at all, 10 = the greatest conceivable burden) were additionally used to assess perceived distress. A 10-point NRS (1 = very poor quality of life, 10 = best possible quality of life) was used to assess quality of life. The NRS is considered a valid tool for measuring the intensity of subjective experiences [27, 28]. The interviews were conducted either via online meetings or by telephone without the presence of third parties and were audio-recorded. Interviews with patients lasted approximately 90–120 min, whereas supplementary interviews with gynecologists lasted approximately 45–60 min. Sample size was not fixed a priori but determined based on data saturation [29]. The point at which the interviews no longer yielded new thematic content was defined as data saturation. Saturation among affected individuals was considered reached at interview 16 and was confirmed through four additional consecutive interviews (17–20) in which no new topics emerged; recruitment was terminated at this point. Data saturation was assessed exclusively for the sample of affected individuals and was not achieved, nor intended to be achieved, in the supplementary sample of five gynecologists, whose interviews served a contextualizing rather than a standalone thematic function.

All interviews were conducted by the first author Katharina Weiss (KW), a female health scientist experienced in qualitative interview research, as part of her doctoral project. KW is a certified trainer in stress and resource management and completed training as a mental health first responder at the Central Institute for Mental Health in Mannheim [30] prior to data collection. There was no prior relationship between the interviewer and the study participants. Reflexivity was addressed throughout both data collection and analysis. As the interviewer’s professional background includes training in stress and resource management, the interview guide and interviewing style were deliberately kept open-ended and non-directive to avoid steering participants toward predefined coping frameworks; participants were encouraged to describe their experiences in their own terms rather than using structured coping terminology. Throughout the data collection period, the interviewer discussed her impressions, assumptions, and emotional responses to the interview content with her supervisor at regular intervals, allowing potential influences on the interview process to be identified and addressed. During data analysis, the coding team (KW, AH, supervisor) engaged in ongoing reflective discussions regarding how their professional backgrounds and prior assumptions about coping and chronic illness might shape category development and interpretation, as described above.

Following completion of the interviews, the audio recordings were pseudonymized and securely stored on a server at Coburg University. The interview transcripts were not returned to participants for review, because the authenticity of the interviews should be preserved [31].

### Data analysis and reliability

The interviews were transcribed in accordance with the guidelines by Dresing and Pehl [32]. Colloquial language and dialectal expressions were standardized during transcription. Based on the interview guides, deductive category systems were initially developed and subsequently expanded through inductive categories during the coding process (see electronic supplement 1). The interview material was coded iteratively using MAXQDA version 26.0.0, and multiple coding was permitted. To enhance coding consistency, the consensus-based coding procedure recommended by O’Connor and Joffe [33] was applied, 25% of the material was double-coded, within the 10–25% range they identify as typical for datasets of this kind. First, one interview was independently double-coded to identify and resolve potential misunderstandings within the coding system. Secondary coding was conducted by Annika Hiller (AH), a female health scientist experienced in qualitative interview research and a doctoral candidate who was not involved in data collection. Subsequently, a further portion of the total interview material was independently double-coded, for a cumulative total of 25%. Rather than calculating a formal quantitative intercoder reliability statistic, each code assigned during double coding was discussed in detail between both coders (KW, AH) until full consensus was reached, and the coding system was revised accordingly. This consensus-based approach was chosen over statistical agreement measures (e.g., Cohen’s Kappa) because it allowed for immediate resolution and joint reinterpretation of ambiguous codes, rather than merely quantifying disagreement [33–35]. The final coding of the complete dataset was then conducted by one researcher (KW).

In the following step, a summarizing and content-structuring content analysis was performed to condense and organize the data. The analysis followed the general qualitative content analysis framework proposed by Mayring [34] as well as Kuckartz and Raediker [35]. Results were presented in a thematic-descriptive manner.

To ensure methodological rigor, the analysis and presentation of findings were guided by established quality criteria proposed by Mayring [34], based on Krippendorff’s standards for content analysis, the classical criteria of reliability and validity in qualitative research, and the eight “Big Tent” criteria for excellent qualitative research proposed by Tracy [36] .

Through regular discussions among team members (KW, AH, Karin Meissner) throughout the data analysis process, the team engaged in self-reflection regarding the extent to which the team members’ identities might have influenced the data analysis. As part of these discussions, all coding decisions made by the team members were also reviewed and discussed. These reflective practices ensured that the team members’ personal experiences did not influence the interpretation of the participants’ experiences. Involving supervisors throughout the entire data analysis process ensures the reliability of the approach and the credibility of the results. Formal member checking, i.e., returning transcripts or findings to participants for review, was not conducted, as the interview transcripts were not returned to participants to preserve the authenticity of their original accounts [31]. Instead, trustworthiness of the thematic findings was supported through data triangulation, combining the perspectives of affected individuals with those of gynecological specialists, allowing subjective patient experiences to be contextualized against professional accounts of gynecological care, as well as through the reflective and consensus-based coding procedures described above.

## Results

### Characteristics of the sample

Interviews were conducted with 20 individuals who had been diagnosed with adenomyosis and involuntary childlessness. Data saturation was reached after the 16th interview, a finding that was confirmed by conducting four additional interviews. The interviews lasted an average of 80 min (SD = 32 min). Sociodemographic characteristics of the participants at the time of data collection are presented in Table 1. The average age was 37.6 years (range: 30–50 years). Fifteen participants were from Germany, two lived in Austria and three in Switzerland. The most were employed full-time and held a university degree. The majority of the participants were married. All women were diagnosed with adenomyosis, thirteen of them with concomitant endometriosis. In 15 participants, the diagnosis was made via laparoscopy; in 2, it was made via transvaginal ultrasound. One person received the diagnosis following a hysterectomy. In 2 cases, the medical report did not include any information regarding the diagnostic procedure.

**Table 1:** Sociodemographics, diagnosis, type of diagnostic procedure, diagnostic delay and NRS scores of the participants at the time of data collection; *n* = 20

ID	age	current employment status	highest level of vocational education	marital status	diagnosis, as documented in the medical report	type of diagnostic procedure	diagnostic delay (in years)	NRS Stressor Adenomyosis (0–10)	NRS Stressor Involuntary Childlessness (0–10)	NRS Quality of life (1–10)
B1	40	full-time employed	State Examination	married	ENZIAN B2/1 FA #ENZIAN P3 O1/0 T0+/1 + B2/1 FA rASRM II	laparoscopy	N/A	7–8	6	5
B2	42	part-time employed	Bachelor’s degree	married	adenomyosis uteri	TVUS	7	7–8	8	N/A
B3	36	unable to work	Master’s degree	unmarried, in a relationship	#ENZIAN A3, B2, C1, P2, T1, adenomyosis uteri	hysteroscopy, laparoscopy	N/A	8	8	5.5
B4	32	full-time employed	Master’s degree	married	adenomyosis uteri PCOM	TVUS	20	6	10	6
B5	38	temporarily part-time employed	Diploma	married	adenomyosis uteri	no information provided	26	6	10	1
B6	32	part-time employed	Bachelor’s degree	married	#ENZIAN P1, O0/0, T3m/3m, A0, B0/2, C0, FA rASRM IV N80.0	hysterectomy	N/A	2	7	8
B7	40	full-time employed	Magister degree	married	#ENZIAN FA	hysteroscopy, laparoscopy	N/A	4	7	7
B8	31	full-time employed	Doctoral degree	unmarried, in a relationship	#ENZIAN P2 O1/0 A3 B3 FA rASRM III	laparoscopy	3	5–6	7–8	8–8
B9	36	full-time employed	Master’s degree	unmarried, in a relationship	#ENZIAN (s) P3 O2/0 T3/3 B2/2 FA FU FI rASRM III	no information provided	25	5–6	6	8–8
B10	44	full-time employed	Master’s degree	married	adenomyosis uteri	TVUS, laparoscopy	N/A	5	N/A	N/A
B11	33	full-time employed	Master’s degree	married	ENZIAN FA	hysteroscopy, laparoscopy	10	6	2–3	7
B12	41	full-time employed	State Examination	married	#ENZIAN P2 O0/0 T0/2 A0 B0 C0 FA rASRM III	TVUS, laparoscopy	26	8	6–7	6
B13	50	full-time employed	Master’s degree	unmarried, in a relationship	adenomyosis uteri rASRM II	hysteroscopy, laparoscopy	N/A	0	0	8
B14	46	full-time employed	Diploma	married	ENZIAN C1 rASRM I adenomyosis uteri	laparoscopy	21	5	0	7
B15	30	unable to work	Teaching	married	#ENZIAN P1 O1/0 T+/+ FA	hysteroscopy, laparoscopy	10	7	5	10
B16	31	unable to work	Teaching	married	#ENZIAN P2, O1/0, cFA	laparoscopy	17	8–9	5	4–5
B17	37	full-time employed	Teaching	separated	ENZIAN P3 O0/1 T3/3 B2/1 C2-3 FA FO	laparoscopy	14	5	3	6
B18	34	full-time employed	Master’s degree	married	#ENZIAN (s) P3 O2/2 T3/3 (-/+) B3/3 C2 FA FI (Appendix) rASRM IV	laparoscopy	N/A	8	6	4
B19	38	full-time employed	Master’s degree	married	#ENZIAN P3 A1 B1/2 V.a. FA T0-/0+ rASRM II	hysteroscopy, laparoscopy	3	1	1	8
B20	41	full-time employed	Bachelor’s degree	married	adenomyosis uteri	TVUS, laparoscopy	2	8	8	10

*Abbreviations*: *n* sample size, *N/A* not applicable, *NRS* numeric rating scale, *PCOM* polycystic ovarian morphology, *rASRM* revised score from the American Society for Reproductive Medicine (a point-based system for classifying the severity of endometriosis during surgery), *TVUS* transvaginal ultrasound examination

The sample of medical professionals comprised five specialists in obstetrics and gynecology from Germany with expertise in the field of adenomyosis who agreed to be interviewed. At the time of the interviews, two participants were employed at certified endometriosis centers, whereas three worked in private or group practices. Three participants were female and two were male, with ages ranging from 40 to 60 years. Their expertise in adenomyosis was acquired through their medical education, specialized training, and extensive clinical experience in the field. The average interview duration was 43 min (SD = 23 min).

### Coding schemas

Based on the interview guides, deductive, guideline-based category systems (coding system for affected individuals see Table 2; complete coding systems for both groups are provided in electronic supplement 1). During the initial coding process, the category systems were further expanded through the addition of inductively derived categories.

**Table 2:** Coding system for affected individuals

Topic	Main Categories
Topic 1: Adenomyosis	MC 1.1: Initial diagnosis
	MC 1.2: Medical information and education after diagnosis
	MC 1.3: Counseling after diagnosis
	MC 1.4: Recommendation of assistance and support services
	MC 1.5: Course of treatment
	MC 1.6: Comprehensibility of information, explanation, and counseling
	MC 1.7: Personal initiative
	MC 1.8: Current situation regarding adenomyosis
	MC 1.9: Peri- and Menopause (inductive)
	MC 1.10: Self-directed learning
Topic 2: Adenomyosis and involuntary childlessness	MC 2.1: Education on infertility risk following a diagnosis of adenomyosis
	MC 2.2: Counseling on achieving pregnancy
	MC 2.3: Course of infertility and desire to have children
	MC 2.4: Current Status: Adenomyosis, Involuntary Childlessness, and Fertility Treatment
	MC 2.5: Recommendations for assistance and support services
	MC 2.6: Current situation regarding acceptance
	MC 2.7: Lack of grandchildren (inductive)
Topic 3: (Stress) burden due to adenomyosis and/or involuntary childlessness	MC 3.1: Stressful experiences
	MC 3.2: Adenomyosis as a stressor (NRS)
	MC 3.3: Stressor: Involuntary Childlessness (NRS)
	MC 3.4: Other current stressors
Topic 4: Coping	MC 4.1: Awareness of the importance of (stress) coping
	MC 4.2: Counseling on (stress) management
	MC 4.3: Relaxation Techniques Used
	MC 4.4: Applied coping strategies
	MC 4.5: Effectiveness of coping
Topic 5: Quality of life (overall; NRS)	
Topic 6: Equity of opportunity and stigma	MC 6.1: Equal opportunities
	MC 6.2: Experienced Stigmatization
Topic 7: Needs and Requirements	MC 7.1: Subjectively Unmet Needs
	MC 7.2: Subjective Assessment of Needs
	MC 7.3: Subjective Spontaneous Ideas
Topic 8: Miscellaneous (inductive)	MC 8.1: Current supply situation
	MC 8.2: RAHT/Rehabilitation stays
	MC 8.3: Relationship
	MC 8.4: Psychotherapy
	MC 8.5: Perceived Medical Invalidation
	MC 8.6: Sexuality
	MC 8.7: Self-efficacy (experiences)

*Abbreviations*: *ART* Artificial Reproductive Technologies, *MC* Main Category, *NRS* Numeric Rating Scale, *RAHT* Rehabilitation after Hospital Treatment

#### Coding system for affected individuals

The coding system for interviews with the affected individuals comprised seven predefined thematic areas and one additional inductively developed area labeled “Other.” Overall, 38 main categories and 62 subcategories were identified. Across the complete interview material, a total of 1,496 codes were assigned.

#### Coding system for gynecologists

For the healthcare professionals, six thematic areas were predefined, with a seventh inductively developed thematic area added for “Other”. Using a deductive, guideline-based approach, 30 main categories were derived, and a total of 123 codes were assigned.

The results presented below are a selection of the overall dataset and are guided by the central research questions of the study.

### Content analysis: Affected individuals

#### Topic 1) Adenomyosis

##### Initial diagnosis and current care situation (RQ1, RQ2)

In the present sample, the diagnosis of adenomyosis was frequently characterized by significant delays. Participants commonly reported that their symptoms had repeatedly been dismissed as “normal menstrual pain.” In many cases, adenomyosis was diagnosed only during further diagnostic procedures, such as fertility treatment, surgery, or as an incidental finding, requiring affected individuals to take considerable personal initiative in pursuing a diagnosis.

“(…) and since then I’ve had a confirmed diagnosis of endometriosis, but, well, I was already thirty-four by then, more or less, but, well, my suffering, my pain definitely started when I was a teenager, around fifteen or so, because my mother had to pick me up from school regularly back then, since it always got so bad for me that I couldn’t sit, stand, or lie down, and I would always end up vomiting.” (B12_042025, lines 45 ff.)

Based on information regarding menarches, symptom onset, and time of diagnosis, the median diagnostic delay was 14 years (IQR: 7–21; *n* = 13). Twelve of 18 respondents reported symptom-onset during menarche or puberty (≤ 20 years of age), including five participants who experienced symptoms immediately at menarche and eight between the ages of 15 and 20.

Reported symptoms covered a broad spectrum and included severe labor-like menstrual pain, heavy bleeding, pain during bowel movements, back and leg pain, gastrointestinal complaints, fatigue, and psychological distress, with pain intensity frequently resulting in significant limitations in daily functioning, including temporary inability to attend school or work. Conventional analgesics (e.g., ibuprofen, acetaminophen) were frequently described as insufficiently effective, while non-pharmacological approaches, such as heat therapy and dietary modifications, were commonly used as supportive measures.

“(…) Well, I always thought that childbirth couldn’t possibly be more painful than what I’m experiencing now. I also thought—since it had never been diagnosed before—that it was either all in my head; actually, most of the time it was considered all in my head, because everyone has their periods and it hurts for everyone. So, the answer was just that I’m extremely sensitive to pain.” (B5_032025, l. 92 ff.)

Participants described that symptoms were frequently normalized or downplayed, both within family contexts and by healthcare professionals, leading to feelings of not being taken seriously; in some cases, symptoms were interpreted as psychosomatic or attributed to nonspecific causes such as body weight, and participants also reported dismissive responses when they raised suspected diagnoses themselves.

“(…) And I was sitting there and she said, and I just asked, I said something like, ‘Could this possibly be endometriosis?’ And she looked at me and then, really, she threw her head back and started laughing out loud. So, I thought, okay, and then she looked at me and said, ‘No, you don’t have endometriosis. It’s so, so rare; you don’t have it.’ Yeah, and somehow a part of me just broke inside, because I thought, well, maybe I really don’t have anything, right?” (B16_052025, lines 119 ff.)

Diagnoses were primarily established in specialized facilities (e.g., endometriosis centers, fertility clinics), whereas diagnosis within primary gynecological care appeared comparatively rare. An unfulfilled desire to have children often acted as a trigger for more extensive diagnostic evaluation and increased medical attention. Participants repeatedly emphasized the importance of their own initiative in obtaining a diagnosis.

“Yes, exactly. Because it’s awful not knowing what’s wrong with you. So, when I finally got a proper diagnosis for the first time, it was like, okay, now at least I have something to go on, a term of some sort.” (B10_042025, lines 615 ff.)

Perceived medical competence varied considerably, with participants reporting misdiagnoses (e.g., fibroids), insufficient information provision, and limited sensitivity toward subjective pain experiences and their consequences for everyday life. Specialized centers were generally perceived as more competent and education-focused, though psychosocial aspects were addressed only to a limited extent even there.

Overall, healthcare provision was frequently perceived as fragmented, reflected in frequent physician changes, long waiting times, and redundant history-taking. Participants’ reliance on self-help groups, social media, and independent research, alongside the financial and personal burden of managing their own care, is addressed in detail in Section "Personal initiative and self-education (RQ2, RQ3, RQ4)".

##### Medical information and education, counseling, and recommendations for support services following diagnosis (RQ3, RQ4)

Following diagnosis of adenomyosis, most participants reported receiving insufficient and often superficial information about the condition, frequently limited to written materials or brief verbal explanations, in some cases delivered by nursing staff without specialized expertise. Communication with physicians was often described as brief, lacking empathy, or entirely absent. Many participants stated that they developed a basic understanding of the condition only through repeated inquiries or independent research. According to participants, important topics such as potential effects on fertility, the chronic course of the disease, or available treatment options were rarely addressed. In several cases, the diagnosis was communicated immediately after surgery while patients were still affected by anesthesia or medication.

“(…) actually, the final diagnosis was given to me postoperatively; I was still feeling a bit delirious at the time. I was told, ‘You have endometriosis - did you ever think that might be it?’ And I thought to myself, ‘What the hell is that?’ I’d heard of it before, but I just thought, ‘Okay, fine. I was glad that nothing malignant had come up. And, exactly, then I was told the diagnosis, and then I did the classic thing and asked Dr. Google, ‘What is that and what does it mean?’” (B6_042025, line 72 ff.)

A minority of participants reported positive experiences in specialized centers or with individual physicians, where communication was perceived as empathetic, participatory, and professionally nuanced.

Counseling regarding available assistance and support services was largely absent; advice concerning lifestyle factors (nutrition, physical activity, stress management) and supplementary therapeutic approaches (physical therapy, osteopathy, complementary therapies) was rarely provided.

“(…) And those are the kinds of things where I think it wasn’t made easy for us. Or what also annoyed me was that, after the surgery, I should have applied for a disability ID card. Nobody told me that. (…)” (B13_042025, line 340 ff.)

Recommendations for additional support services, such as referral to endometriosis centers, psychological counseling, or rehabilitation programs, were reported only sporadically. Overall, medical communication was primarily focused on pharmacological or hormonal treatment approaches (see section "Personal Initiative and Self-Education (RQ2, RQ3, RQ4)").

##### Personal initiative and self-education (RQ2, RQ3, RQ4)

Many participants reported independently requesting referrals to specialized endometriosis centers, fertility clinics, or laparoscopic procedures from gynecologists in outpatient care, as such recommendations were often not provided proactively. Similarly, resources such as the Endo-App^®^, rehabilitation programs, or applications for a disability identification card were frequently pursued on the patients’ own initiative, sometimes despite discouragement or limited knowledge among physicians. Exchanges within self-help groups and anonymous social media communities were described as central sources of both information and emotional support. Frustration with insufficient medical support led many participants to engage in extensive independent research. Frequently mentioned sources included internet searches, social media platforms (especially Instagram), endometriosis associations, specialist books, scientific publications, and patient networks. Participants sought information on symptoms, treatment options, nutrition, lifestyle modifications, and complementary therapeutic approaches such as osteopathy, acupuncture, or Traditional Chinese Medicine (TCM). In some cases, participants reported having developed substantial disease-specific knowledge, which they perceived as exceeding that of some healthcare professionals. Criticism was particularly directed toward the predominant focus of treating physicians on hormonal therapies, while non-pharmacological and complementary approaches were often neglected.

##### Current symptoms and treatment of adenomyosis (RQ7)

Many participants reported severe pain and heavy menstrual bleeding as the predominant symptoms if adenomyosis. Pain was often described as cyclical and fluctuating in intensity, although many participants also experienced a persistent underlying level of pain. Several individuals reported pain severity of such severity that it substantially impaired daily functioning, while conventional analgesics were often perceived as insufficiently effective. Menstrual bleeding was frequently described as excessive and was associated with secondary complications such as iron deficiency. Gastrointestinal complaints, fatigue, and exhaustion were also commonly reported and could severely impair everyday life.

Hormonal therapies were frequently used to reduce pain and bleeding, though not all participants tolerated these treatments well or perceived them as beneficial. Some used high-dose analgesics such as ibuprofen, naproxen, or opioids, and some experimented with complementary or alternative approaches with varying perceived effectiveness. Treatment decisions were strongly influenced by the desire to have children, as hormonal therapies were often perceived as conflicting with fertility goals.

“(…) And, right, and then I took the pill - that was in mid-August, exactly. I’ve been taking it again ever since, because I figured I wouldn’t get pregnant anyway; it’s the wrong time again. And just to hit the pause button, so to speak. And I have to say, I’m feeling better now after seven months. The first few months were rough because I still got my period. I still had the pain, even though I was taking the pill. So, it was kind of doubly bitter. But now I realize I can wear pants again. That was just completely impossible before, because with all that, it just wasn’t working anymore. I don’t have as much pain anymore; I can exercise again. It just kind of clashes a bit with wanting to have kids, right? It’s a bit of a dilemma. But that was important because, well, I had to get back on my feet first.” (B3_032025, l. 182 ff.)

Psychological distress such as anxiety and depressive symptoms was frequently described as a consequence of chronic pain and prolonged medical histories. Participants also linked their care experience to a broader absence of holistic or multimodal treatment approaches, focused instead on individual medical specialties, which they experienced as a further barrier to adequate care.

#### Topic 2) Adenomyosis and involuntary childlessness

##### Information and counseling on infertility risk following diagnosis of adenomyosis (RQ3, RQ4)

Communication regarding the potential impact of adenomyosis on fertility was often described as inconsistent and highly dependent on the individual physician. Many participants reported receiving insufficient information about the possible effects of adenomyosis on fertility and family planning. When information was provided, discussions often shifted quickly toward assisted reproductive technologies without adequately addressing alternative options or broader aspects of reproductive health. While some physicians did not clearly communicate potential fertility-related risks, others advised patients to become pregnant as quickly as possible to alleviate symptoms of adenomyosis.

Many participants reported having to rely on independent research, including internet sources and specialist books, to understand the relationship between adenomyosis and infertility. Fertility clinics were at times perceived as strongly business-oriented and lacking sufficient empathy toward patients’ emotional needs, a pattern reflected in the limited psychosocial support described in 2.3. Participants expressed a desire for comprehensive and transparent information regarding the statistical chances of success, potential risks, and financial burden associated with fertility treatments. In addition, they emphasized the importance of healthcare professionals being adequately trained in communicating with and supporting patients experiencing infertility-related psychological distress.

##### Current situation: Adenomyosis, involuntary childlessness, and the desire to have children (RQ6, RQ7)

The participants described experiences ranging from deep disappointment and traumatic loss to unexpected, spontaneous pregnancies. Many women reported a persistent, unfulfilled desire to have children and a lengthy search for possible treatment options; at the time of the interview or prior to it, 16 participants were undergoing fertility treatment. This had been preceded by 12 months of regular, unprotected sexual intercourse without a natural pregnancy occurring or with experiencing miscarriages. Three participants had become pregnant naturally before being diagnosed with adenomyosis.

Several women underwent assisted reproductive technologies (intrauterine insemination, in vitro fertilization (IVF), intracytoplasmic sperm injection (ICSI)); with the exception of one participant, these treatments were ultimately unsuccessful, and one participant reported severe complications following three ICSI attempts. Other women decided against further fertility treatment because ovarian stimulation was unsuccessful or because the hormonal burden was perceived as too distressing.

Miscarriages were reported frequently and predominantly experienced as traumatic events, with substantial variability in the quality of care received following pregnancy loss, while one participant reported being placed in a maternity ward surrounded by newborns and new parents after her miscarriage, another described receiving highly empathetic and supportive care, including information about burial rights.

##### Recommendations for assistance and support services (RQ3, RQ8)

Many participants reported receiving little or no psychosocial support during fertility treatment, with psychological distress associated with treatment failure and miscarriage frequently managed independently.

“No, not at all, none of that. But I have to say that I then sought out support on my own. No, I mean, after I’d gone through IVF and that, and then had that early miscarriage, I reached a point where I said, ‘I’m not going to keep going alone from here on out.’ By then, I think we’d already spent two years trying inseminations (…) and just processing that was a challenge in itself, but unfortunately, the fertility clinic didn’t offer anything to help with that. So, I had to organize that on my own.” (B7_042025, line 231 ff.)

Only isolated examples of structured psychosocial support within fertility clinics were described.

“Yes, well, and I suppose because the fertility clinic also has its own psychologist on site. And I’ve already seen her (…) Because I was at another fertility clinic before and I know from a friend that many clinics don’t have a psychologist affiliated with them at all. Yes, it’s so very focused on the physical aspect, and I think that’s a huge step forward here - that, in principle, there’s actually someone you can turn to for advice or with whom you can book an appointment. (…)” (B5_032025, line 70 ff.)

As in other areas of care, many participants sought support independently through self-help groups, social media communities, or privately financed therapies. Contact with individuals in similar situations was commonly described as emotionally supportive and helpful in reducing feelings of isolation.

#### Topic 3) Burden and psychological stress

##### Stressful experiences (RQ6)

Experiences related to fertility treatment were frequently described as highly stressful and, in some cases, traumatic. Several participants reported repeated miscarriages, including pregnancy losses following assisted reproductive treatment, which were experienced as emotionally traumatic. Even early pregnancy losses were described as highly distressing and difficult to cope with. In addition, participants perceived societal attitudes toward childlessness as stigmatizing and emotionally burdensome, creating further pressure and feelings of inadequacy.

The psychological burden associated with the unfulfilled desire to have children and the accompanying fears was considerable and, combined with the chronic pain caused by adenomyosis, affected nearly all areas of daily life.

“Exactly, after things got really, really bad last year and I was also under a lot of stress at work, and then I ended up in this acute care hospital, in the psychosomatic ward, partly because of severe depression, because I just couldn’t bear the pain anymore. I just couldn’t go on. I didn’t want to live anymore. That was it, I just didn’t see any point anymore. I realized, I just felt, after more than five years, that the hope of having a child - I don’t know - is somehow gone. Because of that, I somehow feel like I don’t need to work anymore, because what do I need money for if I don’t have children? (…)” (B3_032025, l. 178 ff.)

##### Adenomyosis as a stressor (RQ6)

During the interviews, participants were asked to rate the current severity of burden they experienced in the context of living with adenomyosis using a numeric rating scale ranging from 0 (“no burden at all”) to 10 (“the greatest conceivable burden”). Overall, participants reported a considerable burden, with a median score of 6.0 (IQR: 5.0–7.625; n = 20; see Table 1).

##### Involuntary childlessness as a stressor (RQ6)

Participants also rated the current intensity of their distress related to their involuntary childlessness on the same 0–10 scale. The burden associated with an unfulfilled desire to have children varied considerably among participants, ranging from 2 to 10, with a median score of 6 (IQR: 4–7.75; n = 19; see Table 1).

#### Topic 4) Coping

##### Knowledge of the relevance of coping and counseling on coping (RQ3, RQ4)

The connection between physical and psychological factors was generally not adequately explained by gynecologists or healthcare professionals. Some participants reported having learned, through their studies or psychotherapy, how to deal with stress, burden, and pain, thereby developing positive coping strategies. For some, living with a chronic illness represented a major turning point that prompted the active development of coping strategies. During rehabilitation stays or rehabilitation after hospital treatments, participants were educated through lectures addressing topics such as pain memory and the impact of stress on the body. Recommendations included taking regular breaks and practicing relaxation techniques.

“Yeah, well, the rehabilitation after hospital treatment did have lectures. Yeah, definitely. Things like pain memory and stuff. Where they basically made it clear to us, hey, don’t just endure the pain, but take pills early on or something, take painkillers, but also just explain how pain arises and stuff - that was really good.” (B1_032025, lines 484 ff.)

##### Applied relaxation techniques (RQ7)

Participants described various relaxation techniques as helpful for stress reduction, including Progressive Muscle Relaxation (PMR), yoga, meditation, Tai Chi, Qi Gong, mindful movement, and walking. A recurring theme was the importance of listening to one’s own body and taking regular breaks. Specialized yoga programs adapted exercises to physical limitations, while PMR was frequently used to improve sleep. Pilates, breathing exercises, stretching, using a TENS device, and heat therapies such as grain pillows were also perceived as beneficial. In addition, some participants described sound therapy and the use of apps such as the Endo-App^®^, which combines yoga, relaxation exercises, and symptom tracking, as supportive tools. At the same time, some participants reported difficulties integrating relaxation techniques such as yoga or meditation into their daily lives and occasionally experienced them as an additional source of stress.

##### Coping strategies, relationships, and psychotherapy (RQ7, RQ8)

**Fig. 1:**
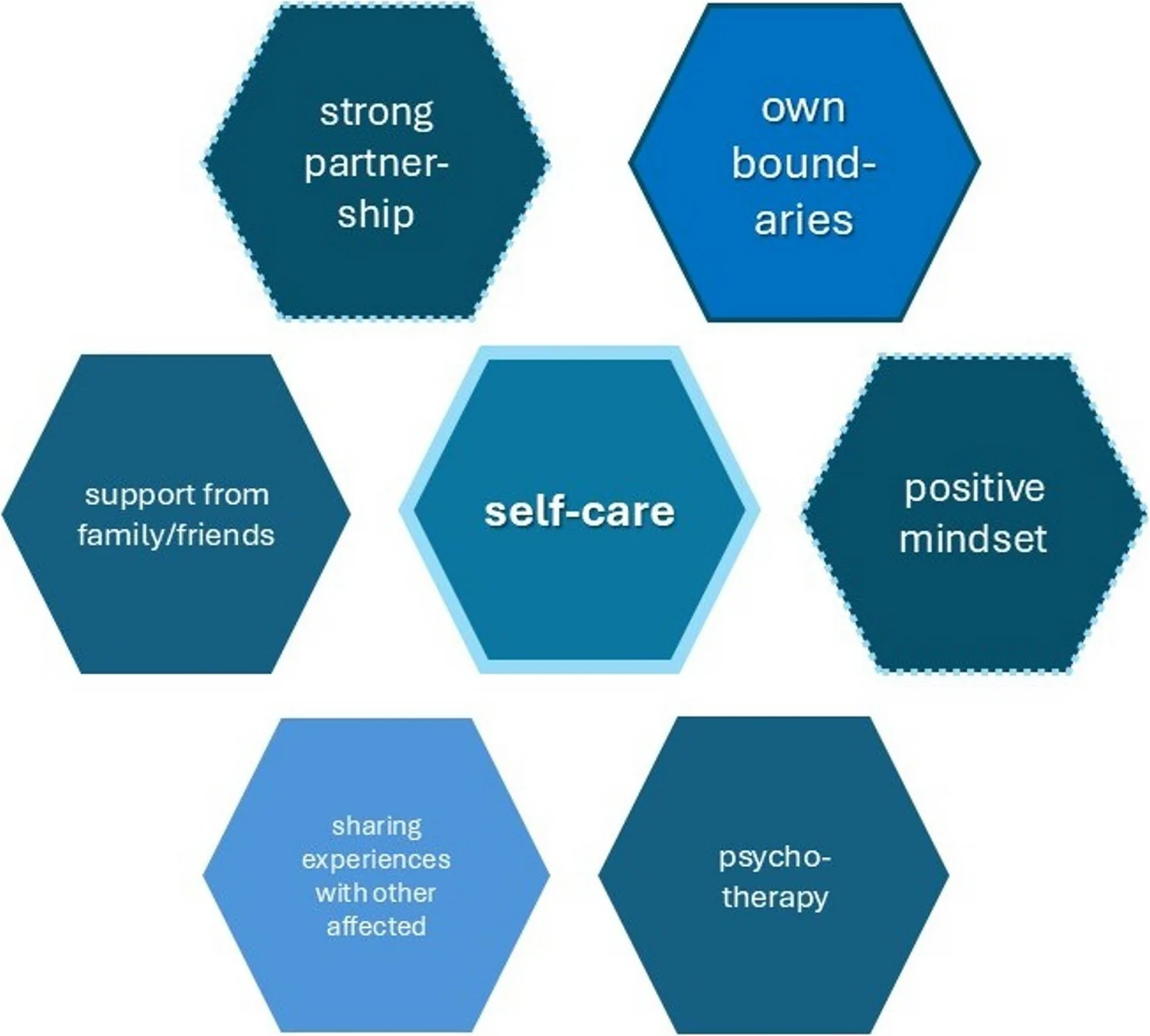
Key coping strategies of those affected (own illustration). Note: The font size, font weight, fill color, and frame illustrate the frequency of mention (e.g. “self-care” was mentioned most frequently)

Most interviewees reported having engaged intensively with their situation and consciously developed strategies to cope with both the psychological and physical burden of the disease. Their coping strategies were diverse (see electronic supplement 2). The most important coping strategies described by participants included self-care, setting boundaries and consciously “saying no,” maintaining a positive mindset, supportive relationships with good communication, support from family and friends, psychotherapy, and sharing experiences with others in similar situations (see Fig. 1).

Self-care included lifestyle changes such as mindful eating (vegetarian/vegan), physical activity, relaxation techniques, and boundary-setting. Social support from partners, friends, and self-help groups helped reduce feelings of isolation. Supportive partnerships provided were described as an important coping resource. Emotional closeness, practical support (e.g., household chores, visits to doctors), and jointly coping with involuntary childlessness often strengthened relationships.

“No, I would definitely say that NOW, above all, we’re pulling in the same direction. I mean, I think there was a time when I was just trying and, yeah, forcing everything somehow. And I do have the feeling that he didn’t want it that way 100%, so forced. But he’s always been the type to say that no matter what I decide - because, as he puts it, I’m the one who’ll suffer the most - no matter what I decide, he’ll stand by me in it. So, he can also imagine a life without children with me.” (B15_052025, lines 226 ff.)

Overall, participants described these strategies as contributing to improved quality of life, greater self-awareness, and increased acceptance, including, in some cases, acceptance of a life without children.

Psychological and emotional support played a particularly central role. Psychotherapy was repeatedly described as central in helping participants process trauma, reduce anxiety, and promote self-acceptance, often over extended periods or during times of crisis. Several participants reported that therapy helped them recognize personal limits and understand themselves better. A confirmed diagnosis was described as especially important, as it provided validation, reduced uncertainty, and enabled more structured coping with the disease.

“If I hadn’t worked so much on myself psychologically over the past year to cope with the setbacks I’ve had, to even process this diagnosis and so on. If I hadn’t really taken the time to think to myself, okay, just being completely honest, okay, maybe I’ll never have a child, and I still want to lead a happy, fulfilling life with a high quality of life, then I have to - and this is what I’ve decided now - simply continue to develop professionally or focus on that, and I’ve just done that, even though it’s actually the most heartbreaking moment imaginable.” (B5_032025, lines 577 ff.)

#### Topic 5) Quality of life (RQ6, RQ8)

Participants rated their current quality of life on a numeric rating scale from 1 (‘very poor quality of life’) to 10 (‘best possible quality of life’); ratings were available for 18 participants, with a median of 7 (IQR: 5.625–8; n = 18). Ratings varied widely: while some rated their quality of life as high or even as 10, others described it as severely impaired, assigning ratings between four and five (see Table 1). Despite the burdens associated with the disease, many participants perceived their quality of life as predominantly good, often attributing this to positive aspects such as supportive relationships, friendships, work, or their ability to actively cope with their situation.

### Content analysis: Gynecologists (RQ5, RQ8)

The results of the additional interviews with gynecologists revealed few new or further insights.

According to the interviewees, collaboration with endometriosis centers and referrals from gynecologists with an own practice increased; however, wait times at specialized centers were often long. The role of private practice gynecologists was viewed critically; they often lacked both the time and the expertise to comprehensively diagnose adenomyosis, leading to prolonged suffering.

“You have to admit, the problem is everywhere. General practitioners are very general – yes, most of them, who’ve been out of hospitals for a long time, never have much time for patients. That’s normal; unfortunately, the system encourages this by paying very little, which means they rely on volume rather than quality, and they can’t spend half an hour talking to patients, and even an ultrasound for endometriosis takes time. It’s not just about where the uterus is or where the ovaries are; I have to look carefully, examine all the organs, check the bladder for endometriosis, yes or no, the uterus, between the uterus and the rectum, the ovaries, the vagina itself, and then you have to search a lot. You have to take your time, and they don’t have that; they can’t schedule patients every 40 min the way I do it here. And the patients have a lot of questions, and that’s what’s missing most with them. That’s just how it is, unfortunately. (…) So that means they don’t have the expertise. Because, where I am now, where we also started here with the endometriosis center, I have special courses on ultrasound. Specifically for endometriosis (…)” (M5_062025, lines 43 ff.)

However, the German healthcare system, and particularly the health insurance billing system, was identified as a key structural problem. It does not allow gynecologists with an own practice sufficient time to conduct a proper medical history and examination when adenomyosis is suspected.

In addition, it was emphasized that the overall state of care in gynecology, particularly for endometriosis and adenomyosis, was also rated poorly by the gynecologists surveyed.

“The healthcare situation is bad everywhere.” (M2_052025, line 210)

Since patients with adenomyosis often experience significant distress and a reduced quality of life, psychological support and the teaching of coping strategies were considered crucial to the success of treatment. The willingness to accept a chronic condition and find ways to live with it was described by the specialists interviewed as playing a central role.

Furthermore, most gynecologists supported the implementation of a multimodal care approach for patients.

“(…) Today, I can see a lot using ultrasound. And that allows me to explain a lot, and then I can work out a strategy with her, because, as I said, these patients need a strategy. They don’t just need surgery. (…) That’s just one part of it. They need a plan so they know exactly what to do after the surgery. Because this comes up again and again. (…)” (M5_062025, line 87 ff.)

## Discussion

This qualitative content analysis of interviews with 20 individuals with a medically documented diagnosis of adenomyosis revealed several factors reported in the context of diagnostic delay, including symptom normalization and trivialization, perceived gaps in clinician knowledge, and experiences of misdiagnosis in primary care. Affected individuals primarily suffered from severe pain, hypermenorrhea, involuntary childlessness, and psychosocial distress. Care was frequently perceived as fragmented and inadequate, with participants reporting insufficient information and limited referral to support services. Access to diagnosis, information, and treatment was often described as requiring substantial personal initiative. It should be noted that a substantial proportion of participants presented with concomitant endometriosis, and the diagnostic procedures underlying the documented adenomyosis diagnoses varied across participants. The experiences and burden reported in this study therefore cannot be attributed to adenomyosis alone and should be interpreted as reflecting the lived experiences of a diagnostically heterogeneous sample of individuals with adenomyosis, in many cases in the context of coexisting or suspected endometriosis.

Based on retrospectively reported symptom onset and year of diagnosis, the median interval between first reported symptoms and the diagnosis of adenomyosis was 14 years (IQR: 7–21; *n* = 13). Twelve of 18 respondents who provided information on symptom onset reported experiencing their first symptoms, particularly pain, during adolescence or early adulthood (≤ 20 years). Adenomyosis has traditionally been described as a condition primarily affecting women aged 40–55 [37–40]. However, this observation should be interpreted with particular caution given the retrospective and non-adenomyosis-specific nature of the symptom data reported here. These findings cannot be used to draw conclusions about the age at which adenomyosis itself typically develops, nor do they permit conclusions regarding the age of onset in the broader adenomyosis population. Retrospectively reported symptoms cannot be attributed specifically to adenomyosis, particularly given the frequent co-occurrence of endometriosis in this population; early symptoms may therefore have been related to adenomyosis, concomitant endometriosis, other conditions, or a combination of factors. Nevertheless, the possibility that adenomyosis may already be present during adolescence is supported by recent imaging-based evidence. A systematic review by Wilk et al. [41] identified sonographic or MRI features of adenomyosis in 25–45% of adolescents and young women (12–25 years) presenting with dysmenorrhea, heavy menstrual bleeding, and pelvic pain, with dysmenorrhea being the most frequently reported symptom (81–100%). These findings indicate that features of adenomyosis can be detected in symptomatic adolescents and young women and thus support the plausibility that at least some of the early symptoms reported by our participants may have occurred in the presence of adenomyosis, even though they do not allow the retrospectively reported symptoms in the present study to be attributed specifically to adenomyosis.

Diagnosis was rarely described as resulting from early clinical suspicion; instead, participants frequently reported that diagnosis followed their own persistent efforts to seek further evaluation. This finding is consistent with observations in endometriosis research by Goel et al. [42] and Young et al. [43] and may suggest potential barriers to the early recognition of adenomyosis. Nearly all participants additionally reported experiences they perceived as dismissal or invalidation of their symptoms and described these experiences as adversely affecting their self-image and trust in the healthcare system. This pattern mirrors observations by Cetera et al. [44], who use the term ‘medical gaslighting’ to describe similar experiences reported by patients with endometriosis. We use the more methodologically neutral term ‘perceived medical invalidation’ here, as our data reflect participants’ subjective experiences rather than an independent assessment of clinicians’ intent. Where holistic, multidisciplinary care (e.g., pain centers or specialized clinics) was accessible, participants frequently perceived this care as beneficial and described it as contributing positively to their quality of life. These experiences are consistent with recommendations by Omtvedt et al. [45] and Noditi et al. [46] for interdisciplinary, specialized care, as well as with broader calls for multidisciplinary chronic pelvic pain management [47].

As adenomyosis-specific literature addressing psychosocial experience, coping, and healthcare-related burden remains limited, the following discussion draws, where appropriate, on the more extensive endometriosis literature for contextualization. This reflects the current evidence base and should not be interpreted as implying that adenomyosis and endometriosis are equivalent conditions. Although the two conditions frequently coexist and may share clinical and pathophysiological features, the nature and extent of their relationship remain incompletely understood, and adenomyosis is considered a distinct clinical entity [1–3].

The parallels between the experiences reported in this study and findings from the endometriosis literature may partly reflect the considerable clinical overlap and frequent co-occurrence of adenomyosis and endometriosis, as well as potentially shared pathophysiological features [2, 3]. Both conditions are characterized by chronic pelvic pain, diagnostic delay, and psychosocial burden, and limited clinical awareness and gaps in the evidence base have been reported for both [48, 49]. However, whereas public and clinical awareness of endometriosis has increased substantially over the past decade [50], adenomyosis appears to have received comparatively less attention. Given their frequent co-occurrence, the respective contributions of adenomyosis and endometriosis to the clinical and psychosocial experiences of individuals affected by both conditions remain difficult to disentangle [2]. These findings suggest that adenomyosis may benefit from increased advocacy and awareness, while also warranting condition-specific attention as a distinct clinical entity [1]. Greater attention to adenomyosis may also help clarify whether experiences and burdens previously discussed predominantly in relation to endometriosis are also relevant to individuals with adenomyosis. Beyond endometriosis, the challenges reported in this study, including prolonged pathways to diagnosis, fragmented care, and limited psychosocial support, also mirror findings reported in the context of other chronic gynecological pain conditions, such as chronic pelvic pain [47] and dysmenorrhea [50]. This convergence may point to broader challenges in the recognition and management of chronic, non-malignant gynecological conditions.

Education regarding potential fertility implications in the context of adenomyosis was also perceived as insufficient. Most participants reported learning about a possible association between adenomyosis and fertility only incidentally, often during postoperative consultations, which they described as a challenging context in which to process this information. These findings are consistent with an exploratory survey by the Endometriosis Association of Germany [49] and a qualitative study by Huang et al. [51], which identified gaps in disease-related knowledge as a key barrier to self-management. Limited or delayed provision of fertility-related information may represent an additional source of burden for individuals navigating both a chronic gynecological condition and an unfulfilled desire to have children, experiences that have been associated with anxiety, depression, and social isolation in previous research [52, 53]. It should be noted that the present study focused on an unfulfilled desire to have children rather than requiring evidence that adenomyosis was the cause of impaired fertility. Accordingly, the co-existence of adenomyosis and involuntary childlessness in this sample should not be interpreted as evidence of a causal relationship between the two. Fertility may be influenced by multiple factors, including concomitant endometriosis, and the respective contributions of these factors cannot be determined from the present study.

Structural barriers, including limited consultation time, insufficient reimbursement, and restricted coverage for non-hormonal and complementary therapies, were also reported by the specialists interviewed, who described similar challenges from their professional perspective. These accounts are consistent with broader evidence of systemic barriers in the care of individuals with chronic gynecological pain conditions [54–56]. However, as the physician sample consisted of only five specialists with particular expertise in adenomyosis and endometriosis, these findings should be understood as reflecting specialist perceptions of current gynecological care rather than as an empirical assessment of general knowledge or practice patterns among gynecologists. The study’s findings regarding perceived gaps in clinical awareness and prolonged pathways to diagnosis are therefore primarily grounded in the accounts of affected individuals, with the physician interviews serving a complementary and contextualizing role.

Despite adenomyosis having been described as early as the 19th century [57], important gaps in its scientific and clinical understanding remain. Compared with endometriosis, which has received increasing public and clinical attention [48], adenomyosis appears to remain comparatively under-recognized [18, 51]. Its frequent coexistence and clinical overlap with endometriosis, also reflected in the present sample, may further complicate the recognition and attribution of symptoms to either condition. Finally, no clear country-specific differences emerged across Germany, Austria, and German-speaking Switzerland. Given the qualitative design and the limited and uneven sample across countries, this observation should not be interpreted as evidence of equivalent healthcare structures or experiences across these settings but may indicate that some of the challenges described are not confined to a single national context.

In the context of limited structured psychosocial support, participants described developing a range of individual coping strategies, echoing findings from research on endometriosis [58] and period pain management more broadly [50]. Feeling taken seriously by healthcare professionals and receiving a diagnosis were frequently described as important turning points in participants’ coping processes [59, 60]. Reported key strategies included self-care, boundary-setting, maintaining a positive mindset, stable relationships, social support, psychotherapy, and peer exchange (see Fig. 1), several of which have also been identified among women with endometriosis [61, 62]. The similarities between the coping strategies reported in the present study and those described in the endometriosis literature may point to common features of psychological adaptation to chronic, pain-associated gynecological conditions. However, given the absence of a direct comparison group, no conclusions can be drawn regarding whether coping processes differ specifically between individuals with adenomyosis and those with endometriosis.

Taken together, the findings indicate that individuals with adenomyosis and involuntary childlessness experience prolonged diagnostic delays, fragmented care, and significant psychosocial burden, underscoring the need for holistic, patient-centered care that integrates medical, psychosocial, and reproductive health needs [47].

### Limitations

#### Sample representativeness and selection bias

Due to the small sample size and multiple potential sources of selection bias, the results of this study are neither representative nor generalizable to the broader population of individuals with adenomyosis. Participants were recruited primarily through endometriosis centers, fertility clinics, rehabilitation clinics, social media, and self-help groups; these recruitment channels may have preferentially reached individuals with more severe or persistent symptoms, greater healthcare dissatisfaction, higher engagement in information-seeking or patient advocacy, or particularly salient healthcare experiences, whether positive or negative. Consequently, the frequency and intensity of the experiences reported in this study, particularly negative healthcare experiences, should not be interpreted as representative of the broader population of individuals with adenomyosis. The sample was further characterized by high educational attainment, consistent with evidence suggesting that individuals with lower educational attainment are less likely to participate in emotionally sensitive, interview-based research [63]. Although employment status, educational attainment and marital status were captured (Table 1), income and migration background were not collected. Their potential influence, as well as the transferability of the findings to individuals with fewer socioeconomic resources, migrant populations, or limited access to healthcare, therefore cannot be assessed. As education, health literacy, and financial resources may shape access to coping resources such as psychotherapy, second opinions, and complementary treatments, the coping strategies identified in this study may not fully reflect those available to or used by more socioeconomically diverse population [64]. Response bias, including social desirability bias [65], and recall bias [66], constitute further limitations. Recall bias is particularly relevant to the median interval of 14 years (IQR: 7–21; *n* = 13) between retrospectively reported symptom onset and documented diagnosis of adenomyosis. Reports of symptom onset frequently dated back to adolescence and cannot establish that these early symptoms represented the onset of adenomyosis. Given the frequent co-occurence of endometriosis in this population and in the study sample, early symptoms may have been related to adenomyosis, endometriosis, other conditions, or a combination of factors. The 14-year estimate should therefore be interpreted as the interval between retrospectively reported onset of symptoms and subsequent diagnosis of adenomyosis, rather than as a precise estimate of either adenomyosis onset or adenomyosis-specific diagnostic delay. As discussed above, imaging-based evidence indicates that features of adenomyosis can be detected in symptomatic adolescents and young women; however, this does not permit retrospective attribution of the early symptoms reported by participants in the present study specifically to adenomyosis. Furthermore, participants were recruited across three German-speaking countries, but the sample was unevenly distributed (15 from Germany, two from Austria, three from Switzerland) and not stratified by country. Although no clear country-specific differences emerged in the present analysis, this should not be interpreted as evidence of comparable healthcare structures or experiences across these settings; the limited and uneven sample size across countries did not allow for a systematic cross-country comparison.

#### Interview modality

Interviews were conducted online or by telephone rather than in person. While this approach facilitated participation across geographically dispersed locations, remote interviewing may differ from face-to-face interviewing in terms of interpersonal interaction and the availability of nonverbal cues, particularly during telephone interviews. Given the emotionally sensitive nature of the topic, interview modality may therefore have influenced how participants communicated their experiences.

#### Diagnostic characterization

This study was not designed to differentiate systematically between isolated adenomyosis and adenomyosis with concomitant endometriosis, and participants were not stratified according to the presence or phenotype of coexisting endometriosis. Although concomitant endometriosis was documented in a substantial proportion of the sample, detailed and standardized information on endometriosis phenotype, prior surgical treatment, and #ENZIAN classification was not consistently available for all participants. This is particularly relevant given that recruitment occurred partly through endometriosis-related channels. Consequently, the respective contributions of adenomyosis, concomitant endometriosis, involuntary childlessness, and other potential factors to participants’ reported symptoms, healthcare experiences, psychosocial burden, and coping cannot be disentangled. The findings should therefore not be interpreted as reflecting experiences attributable specifically to adenomyosis. Diagnostic characterization of adenomyosis was also heterogeneous. Although eligibility required a medically documented diagnosis and Table 1 reports the available diagnostic information and procedures for each participant, standardized information on sonographic criteria (e.g., MUSA features), disease phenotype (focal vs. diffuse), extent or severity, and MRI findings was not available. Moreover, consistency in diagnostic assessment across clinical centers could not be verified. The study population should therefore be regarded as clinically and diagnostically heterogeneous, which needs to be considered when interpreting the findings and assessing their transferability to other populations with adenomyosis.

#### Operationalization of infertility

In line with the psychosocial focus of this study, eligibility was based on the presence of involuntary childlessness rather than on a reproductive-medical diagnosis of infertility according to established clinical criteria. This operationalization was chosen to capture the participants’ experiences of involuntary childlessness but does not establish a causal link between adenomyosis and infertility. Other contributing factors, including male-factor infertility, tubal disease, diminished ovarian reserve, reproductive age, concomitant endometriosis, or the duration of attempts to conceive, could not be systematically assessed or excluded. It should be noted, however, that 16 of 20 participants were undergoing or had undergone assisted reproductive treatment, which is typically initiated only after a documented failure to conceive over ≥ 12 months; this suggests that a substantial proportion of participants likely met clinical infertility criteria [10], although this was not systematically verified as part of our eligibility criteria. Detailed reproductive-medical information was not systematically collected within this qualitative, experience-focused study. Accordingly, the findings should be interpreted as reflecting the experiences of individuals with a documented diagnosis of adenomyosis and involuntary childlessness, rather than as evidence of adenomyosis-related infertility or a causal relationship between adenomyosis and infertility.

#### Physician sample

The physician sample consisted of only five specialists with particular expertise in adenomyosis and endometriosis, all practicing in Germany, and cannot be considered representative of gynecologists more broadly or of clinical practice across the DACH region from which affected participants were recruited. The physician interview data should therefore be interpreted as reflecting the perspectives of a small, highly specialized group on current gynecological care and professional awareness, rather than as an empirical assessment of general knowledge or practice patterns among gynecologists. Findings regarding perceived gaps in clinical awareness and prolonged pathways to diagnosis are primarily grounded in the experiences reported by the affected participants, while the physician interviews provide a complementary and contextualizing professional perspective.

#### Coding procedure

To enhance the consistency of the coding process, 25% of the material was independently coded by two researchers. Coding differences were subsequently discussed and resolved through consensus, and the coding framework was refined accordingly. The remaining 75% of the material was coded by a single researcher using the revised coding framework. Thus, independent double coding was limited to a subset of the data, and consistency of coding across the remaining material was not independently assessed. No formal quantitative measure of intercoder reliability (e.g., Cohen’s kappa) was calculated, as coding agreement was established through consensus-based discussion rather than statistical assessment. This approach should be considered when evaluating the dependability of the qualitative analysis.

### Strengths

Semi-structured interviews were well-suited to exploring the sensitive and under-researched topic of living with adenomyosis and involuntary childlessness, as they provided thematic comparability across interviews while allowing sufficient flexibility to capture participants’ individual experiences, perceived burden, and coping strategies in depth. Conducting the interviews facilitated participation across geographically dispersed locations and may have reduced participant barriers for individuals experiencing health-related limitations. The inclusion of both affected individuals and specialist physicians provided complementary perspectives on healthcare experiences and perceived gaps in care, and the physician interviews provided additional professional context for interpreting the experiences reported by affected participants. Furthermore, recruiting participants from three German-speaking countries broadened the range of healthcare contexts represented in the sample, although the study was not designed to identify or compare country-specific differences. Qualitative content analysis provided a transparent framework for organizing and interpreting the interview material. The combination of summarizing and structuring approaches with thematic-descriptive analysis enabled both systematic categorization and a more contextualized representation of participants’ experiences. The organization of the analysis around predefined thematic areas provided a coherent framework while allowing diverse medical, psychosocial, and healthcare-related experiences to be represented.

## Conclusion

Adenomyosis is a common yet comparatively under-recognized chronic gynecological condition. In this study, participants with a medically documented diagnosis of adenomyosis reported prolonged pathways to diagnosis, fragmented care, insufficient information, and limited psychosocial support, while specialists interviewed described complementary structural challenges, including limited consultation time and insufficient reimbursement. Given the frequent presence of concomitant or suspected endometriosis in the sample, these experiences cannot be attributed specifically to adenomyosis but should be understood within the broader clinical context, in which affected individuals experience and navigate their condition. The findings highlight the potential value of more structured, holistic, and patient-centered approaches to care that integrate medical, psychosocial, and reproductive health needs.

Participants’ accounts further indicate a need for accessible psychosocial support, particularly in the context of involuntary childlessness, while the coping strategies identified illustrate the importance of considering psychological and social dimensions alongside medical care. Greater attention to adenomyosis in medical education and specialist training may also contribute to increased clinical awareness.

Taken together, the perspectives of affected individuals and specialists provide an empirical basis for the development and evaluation of more comprehensive support and care approaches for individuals with adenomyosis and involuntary childlessness. Given the qualitative and diagnostically heterogeneous nature of the present study, these findings should be regarded as hypothesis-generating rather than as evidence of the effectiveness of specific interventions or causal relationships. Future research should evaluate the identified care needs and proposed approaches in larger and more diverse samples and examine their effectiveness using appropriate prospective and quantitative study designs.

## Supplementary Information

Electronic supplement 1: Interview guides and coding systems. Part 1: Interview guide for patients and gynecologists. Part 2: Complete coding systems.

Electronic supplement 2: Coping strategies described by participants, n = 20 (own illustration). Note: Multiple answers possible.

Electronic supplement 3: COREQ-Checklist.

## Data Availability

The datasets generated and analysed during the current study are not publicly avail-able, as they contain health and personal data pertaining to a vulnerable patient group, but are available from the corresponding author on reasonable request.
