# Supplementary material for: Living with adenomyosis and involuntary childlessness: a qualitative interview study
Source: BMC Womens Health. 2026 Sep 14;26:455. doi: 10.1186/s12905-026-04880-7 (PMC13573374; doi:10.1186/s12905-026-04880-7)
Supplement: Supplementary file 1 [file 12905_2026_4880_MOESM1_ESM.docx]

**Electronic Supplement 1**

**Part 1**:	Interview Guide for Patients and Gynecologists

**Part 2**:	Complete coding systems

**Part 1:** Interview Guides

**Interview Guide Affected Individuals**

Time frame: 90 minutes

Date:______________

Number: _B___________

1.	Introduction and greeting (1–2 minutes)

2.	Opening question (2–3 minutes)

3.	Main section

| **Main topic** | **Main question** | **Transition** | **Maintenance** | **Time frame (approx.)** |
| --- | --- | --- | --- | --- |
| Adenomyosis / Endometriosis | Diagnostics (duration, procedure):   - How long have you known about your diagnosis? - What symptoms/problems led you to see your doctor for the first time? - How did your doctor respond to your concerns? | - How long did it take from the first symptoms to the diagnosis? - What are your main symptoms right now? |  | 10 min |
|  | Initiative for diagnosis:   - Who took the initiative to seek a diagnosis? - Did you take the initiative yourself to get a diagnosis? - Or did the initiative come from your gynecologist? |  |  |  |
|  | Explanation/medical information after diagnosis   - Were you given information after the diagnosis was made? | If so, about what?   - Clinical picture - Comorbidities - Treatment options - (In)fertility?   If so, by whom?   - A doctor at the hospital? - Referred to a gynecologist? - Nurse? - Medical assistant? - Specialized staff? - Other? | How clear and understandable was the information to you? |  |
|  | Counseling after diagnosis   - Were you informed about support services after the diagnosis was made? - About support services in the non-medical field? | If so, by whom? | How clear and understandable was the information to you? |  |
|  | Current treatment:   - Is your endometriosis/adenomyosis currently being treated?   If so: How? With what?   - Hormone therapy - Alternative therapy - Non-pharmacological therapy   If you have any comorbidities:   - Are these currently being treated? - If so, how/with what? | Who is treating you?   - How well do you feel cared for? - How well do you feel understood? - How well informed do you feel? - How clear and understandable is the information provided by them to you? | How competent do you consider your current doctor to be? |  |
| Involuntary childlessness | To return (once again) to the topic of infertility:   - When you received your initial diagnosis, were you also informed about potential issues regarding your desire to have children? | If so, by whom?  If so, what was explained?  ---------  If not, did you come across this information on your own?  Where?  Through whom? | How clear and understandable was the information to you? | 10 min |
|  | Were you presented with possible options for fulfilling your desire to have children? | If so, what?   - ART - Adoption - Etc.   ----------------  If no:   - How did you find out about this? - Where? - Through whom? | How clear and understandable was the information to you? |  |
|  | Current desire to have children:   - Do you currently wish to have children? - Have you already tried to get pregnant naturally? - Have you had any experience with ART? - If so: Which procedure? - Why? | What emotional stress was this associated with?  What emotional challenges did this involve?  What psychological stress was this associated with? | Would you try again? |  |
|  | Have you ever had a miscarriage? | - How did you cope with it? - How did the people around you cope with it? - How did your treating physician handle it? - How did you cope with the emotional strain? - Have you experienced any stigma related to this? |  |  |
|  | If you had a hysterectomy:   - When, how, and why were you advised to have a hysterectomy? - Were you provided with (outpatient) support services to help you cope? | - What psychological stress was associated with this? - How did you cope with it? - How did you cope with this? - How are you currently dealing with it?   Is there still a desire to have children? |  |  |
|  | Were you offered any advice regarding unfulfilled desire to have children, ART, adoption, etc.  and/or  miscarriage(s)  and/or  regarding acceptance of involuntary childlessness? | - If so, by whom? - If so, which ones? | - Did you take advantage of these? - If so, what? - If so, what helped you? - What didn’t help you? |  |
|  | Do you accept your (current/permanent) involuntary childlessness? | - Self-acceptance? - Acceptance by others (e.g., partner, parents, family, grandparents, friends,…) |  |  |
| Stress | Stressors of adenomyosis/endometriosis:  Everything that affects the body from within and without is stress, including adenomyosis/endometriosis   - How stressed do you currently feel because of your adeno/endo?   Scale 0–10, 0 = not stressed at all, 10 = extremely stressed |  |  | 10 min |
|  | Stressor: Involuntary childlessness:  Everything that affects the body from within and without is stress, including unsuccessful fertility treatment/involuntary childlessness/etc.   - How stressed do you currently feel because of your involuntary childlessness?   Scale 0-10, 0 = not stressed at all, 10 = extremely stressed |  |  |  |
|  | Are there any other sources of stress in your life right now? | If so, what are they?  How severe are they? (Scale 0–10) |  |  |
| Coping | Stress management:  It’s important to manage stress > Coping and coping skills  Are you aware of these connections? | If so, who told you about them?  When?  How? | How clear and understandable was this information to you? | 15 min |
|  | Have you already learned a specific method for managing stress? | If so, which one?  How was that for you?  Do you still use it? If not, why not?  If so, how often?  What has changed as a result? |  |  |
| Well-being | How would you rate your current well-being?  Scale 0–10, 0 = very poor, 10 = very good |  |  | 5 min |
| Quality of life | How would you rate your current quality of life?  Scale 0–10, 0 = very poor, 10 = very good |  |  |  |

4.	Education

5.	Closing Remarks and Prospects

**Interview Guide Gynecologists**

Time slot: 45 minutes

Date:______________

Number: _M___________

1.	Introduction and welcome (1–2 minutes)

2.	Opening question? (2–3 minutes)

3.	Main Body

| **Main topic** | **Main question** | **Transition** | **Maintenance** | **Time frame (approx.)** |
| --- | --- | --- | --- | --- |
| Adenomyosis / Endometriosis | Differentiation:   - Is adenomyosis a distinct condition? - How do you classify it? How do you distinguish endometriosis from adenomyosis? - Or why don’t you distinguish between them? |  |  |  |
|  | Diagnostics (Duration, Procedures):   - How long have the patients you see been experiencing symptoms without a diagnosis? - Why does so much time still pass between the first symptoms and a diagnosis?   Especially for private practice gynecologists:   - Why are private practice gynecologists, in particular, still so unaware of this condition? - Why do most private practice physicians lack sufficient knowledge regarding endometriosis and adenomyosis? - What solutions do you propose? | - What are the main symptoms that patients present with when they see you? - What comorbidities do you frequently observe in connection with endometriosis/adenomyosis? - How many of the patients who come to see you do so on their own initiative? |  | 10 min |
|  | Patient education following diagnosis:   - Do you provide comprehensive information about the condition after diagnosis? | If not, why not?  If yes, about:   - Clinical presentation? - Comorbidities? - Treatment options - Stress - Psychosocial stress?   If so, who?   - You yourself? - Nurse? - Referral to a private gynecologist? - Additional specialized staff? - Other? |  |  |
|  | Counseling after diagnosis:   - Once the diagnosis has been made, do you provide information about available assistance and support services? - About non-medical support services? - On their own initiative or in response to requests from those affected? - About support services in the non-medical field? | If not, why not?  If yes, who?   - You yourself? - A nurse? - A referral to a private gynecologist? - Specialized staff? - Other | Are the services being accepted by those affected? |  |
|  | Documentation in the doctor’s letter:   - While searching for participants for the study: Often, only endometriosis is documented in the doctor’s letter; adenomyosis is mentioned verbally in passing. Why isn’t this also documented? - Does this seem to be a structural “problem” not only in Germany? |  |  |  |
| Infertility | To now turn to the topic of involuntary childlessness:   - After the diagnosis is made, do you also explain potential difficulties and issues related to fertility? - Does the initiative come from you or the patient? | If not, why not?  If so, how?  What information do you provide?  Do you provide information about outpatient assistance and support services? |  | 5 min |
|  | Counseling after diagnosis:   - Do you provide counseling on options for fulfilling a desire to have children? - Does the initiative come from you or the person concerned? | If not, why not?  If yes, how?  What do you convey?  Do you provide information about outpatient assistance and support services? | Are the services accepted by those affected? |  |
| Stress | Counseling after diagnosis  Everything that affects the body from within and without is stress, including endometriosis/adenomyosis (symptoms), unfulfilled fertility/involuntary childlessness, and everything associated with it, as well as any comorbidities:   - Do you provide information about this? - About the relevance? | - Do you provide information on outpatient assistance and support services? |  | 10 min |
|  | Counseling on stress and health   - Do you provide information on stress management strategies? | - Do you offer counseling on your own initiative? - Does the initiative come from the person concerned? - Do you provide information on outpatient assistance and support services? |  |  |
| Endo/Adeno Therapy | In some cases, a hysterectomy is indicated—even if the patient still wishes to have children:   - Do you provide information about outpatient assistance and support services? |  |  |  |
| Competence | Professional expertise:  I asked you earlier why you decided to specialize in the field of endometriosis.   - How often do you pursue continuing education in this area? - Do you conduct your own research in this field? | If so, on what topics? |  | 10 min |

4.	Conclusion

5.	Closing Remarks and Prospects

**Part 2**: Complete Code Systems

**Code System Affected Individuals**

| **Topic** | **Top-level categories** | **Subcategories** |
| --- | --- | --- |
| Topic 1: Adenomyosis | MC 1.1: Initial diagnosis | SC 1.1.1: Diagnosis  Subcategory 1.1.2: Duration/Course  SCK 1.1.3: Symptoms  SC 1.1.4: Treatment (recommendation)  SC 1.1.5: Medical Competence  SC 1.1.6: Initiative for Diagnosis  SC 1.1.7: Diagnostic Procedures  SC 1.1.8: Location of Initial Diagnosis |
|  | MC 1.2: Medical information and education after diagnosis |  |
|  | MC 1.3: Counseling after diagnosis |  |
|  | MC 1.4: Recommendation of assistance and support services | SC 1.4.1: Referral Coordinator  SC 1.4.2: Type of services  SC 1.4.3: Utilization |
|  | MC 1.5: Course of treatment | SC 1.5.1: Facility providing follow-up care  SC 1.5.2: Course of Surgery  SC 1.5.3: History of Non-Surgical Treatment  SC 1.5.4: Success of Further Treatments  SC 1.5.5: Information and Counseling During the Course of Treatment  SC 1.5.6: Doctor-Patient Interaction During Treatment |
|  | MC 1.6: Comprehensibility of Information, Education, and Counseling |  |
|  | MC 1.7: Patient initiative |  |
|  | MC 1.8: Current situation regarding adenomyosis | SC 1.8.1: Current main symptoms  SC 1.8.2: Current treating physician/facility  SC 1.8.3: Current treatment |
|  | MC 1.9: Peri-/menopause (inductive) |  |
|  | MC 1.10: Self-directed learning |  |
| Topic 2: Adenomyosis and involuntary childlessness | MC 2.1: Counseling on infertility following a diagnosis of adenomyosis | SC 2.1.1: Person providing information on infertility  SC 2.1.2: Content of counseling on infertility  SC 2.1.3: Comprehensibility of the information  SC 2.1.4: Self-directed learning  SC 2.1.5: Doctor-patient interaction (inductive) |
|  | MC 2.2: Counseling on Fertility Treatment | SC 2.2.1: Counselor for assisted reproduction  SC 2.2.2: Content of fertility treatment counseling  SC 2.2.3: Comprehensibility of information  SC 2.2.4: Doctor-Patient Interaction (inductive)  SC 2.2.5: Self-directed learning |
|  | MC 2.3: Course of involuntary childlessness | SC 2.3.1: Referral facility  SC 2.3.2: Course of treatment  SCK 2.3.3: Education and counseling during the course of treatment  SC 2.3.4: Doctor-patient interaction during the course of treatment |
|  | MC 2.4: Current Status: Adenomyosis, Involuntary childlessness, and Ongoing Fertility Treatment | SC 2.4.1: Natural conception  SC 2.4.2: ART  SC 2.4.3: Miscarriage(s)/Stillbirth(s)  SC 2.4.4: Hysterectomy due to adenomyosis |
|  | MC 2.5: Recommendation of assistance and support services | SC 2.5.1: Types of services  SC 2.5.2: Referral provider/institution  SC 2.5.3: Utilization of assistance and support services for infertility and ongoing fertility treatment |
|  | MC 2.6: Current situation regarding acceptance |  |
|  | MC 2.7: Lack of grandchildren (inductive) |  |
| Topic 3: (Stress) burden due to adenomyosis and/or involuntary childlessness | MC 3.1: Stressful experiences |  |
|  | MC 3.2: Adenomyosis as a stressor (NRS) |  |
|  | MC 3.3: Stressor: Involuntary childlessness (NRS) |  |
|  | MC 3.4: Other current stressors |  |
| Topic 4: Coping | MC 4.1: Awareness of the importance of (stress) coping |  |
|  | MC 4.2: Counseling on (stress) management |  |
|  | MC 4.3: Applied relaxation techniques | SC 4.3.1: Effectiveness of applied relaxation techniques |
|  | MC 4.4: Applied coping strategies |  |
|  | MC 4.5: Effectiveness of coping |  |
| Topic 5: Quality of life (overall; NRS) |  |  |
| Topic 6: Equity and Stigma | MC 6.1: Equity | SC 6.1.1: In the workplace  SC 6.1.2: in the social and private sphere  SC 6.1.3: in society |
|  | MC 6.2: Experienced Stigmatization | SC 6.2.1: in the workplace  SC 6.2.2: in the social and private sphere  SC 6.2.3: in society |
| Topic 7: Needs and Requirements | MC 7.1: Subjectively Unmet Needs |  |
|  | MC 7.2: Subjective assessment of needs |  |
|  | MC 7.3: Subjective spontaneous ideas |  |
| Topic 8: Miscellaneous (inductive) | MC 8.1: Current Care Situation | SC 8.1.1: Practicing gynecologists with expertise in endometriosis/adenomyosis  SC 8.1.2: Certified endometriosis center  SC 8.1.3: Self-help group  SC 8.1.4: Counseling center  SC 8.1.5: Fertility clinics  SC 8.1.6: Private practice specialists offering multimodal therapy  SC 8.1.7: Responsibility & feeling left alone  SC 8.1.8: Support services for those affected  SC 8.1.9: Health insurance |
|  | MC 8.2: Rehabilitation after Hospital Treatment/Rehabilitation stays | SC 8.2.1: Initiating the application process  SC 8.2.2: Services During the Stay  SC 8.2.3: Doctor-patient interaction  SC 8.2.4: Satisfaction with the Overall Stay  SC 8.2.5: Integration into Daily Life |
|  | MC 8.3: Partnership |  |
|  | MC 8.4: Psychotherapy |  |
|  | MC 8.5: Medical Gaslighting |  |
|  | MC 8.6: Sexuality |  |
|  | MC 8.7: Self-efficacy (experience) |  |

**Code System Gynecologists**

| **Topic** | **Top-level Categories** |
| --- | --- |
| Topic 1: Adenomyosis | MC 1.1: Subjective classification of adenomyosis (entity) |
|  | MC 1.2: Time to diagnosis and diagnosis |
|  | MC 1.3: Main symptoms |
|  | MC 1.4: Comorbidities |
|  | MC 1.5: Initiative on Diagnostics |
|  | MC 1.6: Patient education following a diagnosis of adenomyosis |
|  | MC 1.7: Counseling following a diagnosis of adenomyosis |
|  | MC 1.8: Utilization of non-pharmacological support services |
|  | MC 1.8: Current treatment options for adenomyosis |
| Topic 2: Adenomyosis-induced infertility | MC 2.1: Information regarding potential infertility following a diagnosis of adenomyosis |
|  | MC 2.2: Counseling on fulfilling the desire to have children |
|  | MC 2.3: Providing information on assistance and support services in the outpatient setting |
|  | MC 2.4: Clinical assessment (inductive) |
| Topic 3: Stress caused by adenomyosis and involuntary childlessness | MC 3.1: Education on stress related to adenomyosis |
|  | MC 3.2: Education on stress related to involuntary childlessness/unfulfilled desire to have children |
|  | MC 3.3: Providing information on assistance and support services in the outpatient setting |
| Topic 4: Coping | MC 4.1: Counseling on stress management |
|  | MC 4.2: Counseling on coping with (stress-related) strain (and coping strategies) |
|  | MC 4.3: Providing information on assistance and support services in the outpatient setting |
|  | MC 4.4: Reports on applied methods |
|  | MC 4.5: Reports on the effectiveness of applied methods |
|  | MC 4.6: Other coping strategies |
| Topic 5: Professional Competence | MC 5.1: Degree in Human Medicine |
|  | MC 5.2: Rationale for professional specialization |
|  | MC 5.3: Professional competence |
|  | MC 5.4: Training as a specialist |
|  | MC 5.5: Continuing Education and Training |
|  | MC 5.6: Own Research Activities |
| Topic 6: Healthcare Needs | MC 6.1: Subjective assessment of needs |
|  | MC 6.2: Subjective spontaneous idea(s) |
| Topic 7: Miscellaneous (inductive) | MC 7.1: Current healthcare situation |
|  | MC 7.2: Healthcare system, health insurance companies |
|  | MC 7.3: Subjective assessment of lack of gynecological expertise |
|  | MC 7.4: Psychotherapy |
|  | MC 7.5: Rehabilitation after Hospital Treatment/Rehabilitation stays |
