# Supplementary material for: Living with adenomyosis and involuntary childlessness: a qualitative interview study
Source: BMC Womens Health. 2026 Sep 14;26:455. doi: 10.1186/s12905-026-04880-7 (PMC13573374; doi:10.1186/s12905-026-04880-7)
Supplement: Supplementary file 2 [file 12905_2026_4880_MOESM3_ESM.docx]

**COREQ Checklist**

| **Topic** | **Item No.** | **Guide question/description** | **Reported on Line No.** |
| --- | --- | --- | --- |
| **Domain 1: Research team and reflexivity** | | | |
| *Personal characteristics* | | | |
| Interviewer/facilitator | 1 | Which author(s) conducted the interview or focus group? | 219 |
| Credentials | 2 | What were the researcher’s credentials? E.g., PhD, MD | 219–223  252-254 |
| Occupation | 3 | What was their occupation at the time of the study? | 220  253 |
| Gender | 4 | Was the researcher male or female? | 219  252 |
| Experience and training | 5 | What experience or training did the researcher have? | 220-223  252-254 |
| *Relationship with participants* | | | |
| Relationship established | 6 | Was a relationship established prior to the start of the study? | 223-224 |
| Participant’s knowledge of the interviewer | 7 | What did the participants know about the researcher? e.g., personal goals, reasons for conducting the research | 197-198 |
| Interviewer characteristics | 8 | What characteristics were reported about the interviewer/facilitator? e.g., bias, assumptions, reasons, and interests in the research topic | 854-967 |
| **Domain 2: Study design** | | | |
| *Theoretical framework* | | | |
| Methodological Approach and Theory | 9 | What methodological approach was cited as the basis for the study? e.g., grounded theory, discourse analysis, ethnography, phenomenology, content analysis | 241–287 |
| *Participant selection* | | | |
| Sampling | 10 | How were participants selected? e.g., purposive, convenience, consecutive, snowball | 180-198 |
| Method of approach | 11 | How were participants approached? e.g., face-to-face, telephone, mail, email | 207--208 |
| Sample size | 12 | How many participants were in the study? | 290 |
| Non-participation | 13 | How many people refused to participate or dropped out? Reasons? | 17 (no further response after being informed about the study) |
| *Setting* | | | |
| Data collection setting | 14 | Where was the data collected? e.g., home, clinic, workplace | 199-210 |
| Presence of non-participants | 15 | Was anyone else present besides the participants and researchers? | 208 |
| Description of sample | 16 | What are the key characteristics of the sample? e.g., demographic data, date | 288-302 + Table 1 |
| *Data collection* | | | |
| Interview guide | 17 | Were questions, prompts, or guides provided by the authors? Was it pilot-tested? | 200-202 |
| Repeat interviews | 18 | Were repeat interviews conducted? If yes, how many? | none |
| Audio/visual recording | 19 | Did the research use audio or visual recording to collect the data? | 207–208, 237-240 |
| Field notes | 20 | Were field notes taken during and/or after the interview or focus group? | none |
| Duration | 21 | How long did the interviews or focus group last? | 292-293  312-313 |
| Data saturation | 22 | Was data saturation discussed? | 210-218 |
| Transcripts returned | 23 | Were transcripts returned to participants for comment and/or correction? | 238-240 |
| **Domain 3: Analysis and findings** | | | |
| *Data analysis* | | | |
| Number of data coders | 24 | How many data coders coded the data? | 246-262 |
| Description of the coding tree | 25 | Did the authors provide a description of the coding tree? | 314-328 + Table 2 |
| Derivation of themes | 26 | Were themes identified in advance or derived from the data? | 244-247 |
| Software | 27 | What software, if applicable, was used to manage the data? | 247 |
| Participant verification | 28 | Did participants provide feedback on the findings? | none |
| *Reporting* | | | |
| Quotations presented | 29 | Were participant quotations presented to illustrate the themes/findings? Was each quotation identified? e.g., participant number | 334-708 |
| Data and findings consistent | 30 | Was there consistency between the data presented and the findings? | 334-708 |
| Clarity of major themes | 31 | Were major themes clearly presented in the findings? | 334-708 |
| Clarity of minor themes | 32 | Is there a description of diverse cases or a discussion of minor themes? | 334-708 |
